# Rethinking COVID-19 test sensitivity-a strategy for improving the detection limit

**DOI:** 10.11604/pamj.2021.39.244.30131

**Published:** 2021-08-16

**Authors:** Mohammed Kalim Akhtar, Ross Ka-Kit Leung, Gulfaraz Khan

**Affiliations:** 1 Department of Chemistry, College of Science, United Arab Emirates University, Al Ain, United Arab Emirates,; 2 Stanley Ho Centre for Emerging Infectious Diseases, Faculty of Medicine, The Chinese University of Hong Kong, Shatin, Hong Kong, China,; 3 Medical Microbiology and Immunology, College of Medicine and Health Sciences, United Arab Emirates University, Al Ain, United Arab Emirates,; 4 Zayed Center for Health Sciences, United Arab Emirates University, Al Ain, United Arab Emirates

**Keywords:** SARS-CoV-2, false negatives, diagnostic workflow, real-time polymerase chain reaction

## Abstract

Numerous genetic tests for the detection of the severe acute respiratory syndrome coronavirus 2 (SARS-CoV-2) virus, including those based on the ever-popular real-time polymerase chain reaction (RT-qPCR) technique, have been reported. These diagnostic tests give false negatives particularly during the early and late stages of COVID-19 clearly indicating inadequate test sensitivity. The entire COVID-19 diagnostic workflow is often overlooked and given very little attention. Herein, we propose that volumetric modifications to COVID-19 workflows would significantly improve detection limits. We would therefore encourage researchers to adopt a holistic approach, in which all the steps of a COVID-19 diagnostic workflow, are carefully scrutinised, particularly those upstream factors at the viral sampling and pre-analytical stages.

## Perspectives

Since the onset of the COVID-19 pandemic, a variety of diagnostics tests for detecting the SARS-CoV-2 genome have been reported [1]. Due to the urgent need to adopt approaches that permit rapid identification of infected members of the population, current COVID-19 diagnostic workflows are configured with speed in mind [2]. Protocols have therefore been devised to shorten the sample-to-answer time of the workflow and maximise sample throughput [3,4]. An important aspect of diagnostic testing is the limit of detection. This is the lowest analytical signal that can be detected, one that is distinguishable from an established baseline. Given that COVID-19 patient samples with low viral ribonucleic acid (RNA) loads still yield cultivable SARS-CoV-2 virus, one cannot entirely exclude the possibility that viral transmission could still occur at very early stages of the infection, possibly even below the detection limits of current commercial kits for COVID-19 testing [5-8]. Not only do cost-cutting measures that cheapen test kits or the adoption of test kits that offer poor limits of detection undermine test sensitivity but more importantly, increase the frequency of false negatives, which have in turn markedly hindered our efforts in controlling the spread of SARS-CoV-2 [9,10]. Every 10-fold decrease in diagnostic sensitivity is estimated to result in a miss of one in eight infected patients [11]. Inappropriate healthcare and containment responses due to misdiagnosed individuals will only lead to further spread of SARS-CoV-2.

**COVID-19 diagnostic workflow:** to gain a better understanding of how test sensitivities could be improved, we need to first consider the entire COVID-19 diagnostic workflow which typically consists of three stages: i) sample collection, ii) RNA extraction, and iii) nucleic acid detection [12].

**Stage 1-sample collection:** the nasopharyngeal swab is the most common sampling technique for COVID-19 testing as it attains the highest sensitivity [13]. This technique, which is carried out by a trained health care professional, involves the insertion of a long flexible swab into the nostril along the floor of the nasal cavity and into the nasopharynx. The swab content is then mixed with a viral transport medium (VTM), usually ranging in volume from 1 to 3ml, to preserve the virus and its RNA content. The composition of the medium helps to maintain the integrity of sampled cells, as well as reduce the possibility of contamination from bacteria and fungi.

**Stage 2-RNA extraction:** to ensure the best sensitivities for nucleic acid detection, extraction of the viral RNA is an important pre-analytical step of COVID-19 workflows [14-16]. With the aid of commercial RNA isolation kits, this step essentially removes contaminants and improves the signal-to-noise ratio of the signal generated during stage 3 of the workflow. These kits contain chaotropic agents to precipitate and remove unwanted materials such as RNases that can compromise sample integrity and/or interfere with downstream detection. The viral RNA is bound to a proprietary resin and subsequently eluted in a small volume of buffer. Consequently, this step results in several-fold concentration of the viral RNA.

**Stage 3-nucleic acid detection:** in the final stage of the workflow, the presence of the viral RNA needs to be coupled to a signal output. For diagnostic purposes, fluorescence is generally preferred for ease of probe synthesis and coupling with common experimental steps, as well as allowing detection with the naked eye. For COVID-19 testing, the favoured method for signal amplification is RT-qPCR [14]. The RNA is converted to DNA, via a reverse transcriptase step, followed by up to 40 cycles of temperature-dependent reactions for DNA replication. Each cycle of replication results in an incremental increase of fluorescence over time which can be used to quantitate the amount of RNA in the sample. The run-time for an RT-qPCR analysis can vary from 1 to 2 hours. Though RT-qPCR remains the gold standard for COVID-19 testing, other techniques such as RT-LAMP (loop-mediated isothermal amplification) [17], real-time reverse-transcription recombinase polymerase amplification assay (RT-RPA) [18], and NEAR (Nicking enzyme-assisted reaction) [19], as well as CRISPR-based detection systems are currently being developed [20].

**What is the limit of detection?** when it comes to test sensitivity, much of the focus and attention is given to the underlying technology at the detection stage of the workflow [21,22]. This is understandable considering that important conclusions need to be drawn on diagnostic specificity, a critical parameter that determines whether the RNA in a patient sample truly emanates from the SARS-CoV-2 genome. But from a detection standpoint, how well do the various technologies hold up for COVID-19 diagnostics? As part of an external quality assessment, the Foundation for Innovative New Diagnostics (FIND) tested serval commercial kits, mainly those based on RT-qPCR, under standardized conditions using clinical samples [23]. They found that the majority of commercial RT-qPCR kits offered a detection limit of 0.05-0.1 copy per μl of reaction [23]. Isothermal techniques such as RT-LAMP and CRISPR, on the other hand, have lower detection limits of around 1 copy per 1 μl reaction [15,17]. There is still enormous room for improving the test sensitivity of any COVID-19 diagnostic workflow when one considers the workflow in its entirety rather than just the end point. To understand how this might be achieved, the limit of detection needs to be redefined in relation to the volume of the original patient sample, in other words, the RNA copy number per ml of viral transport medium [24]. Current state-of-the-art workflows, those based on RT-qPCR, are capable of achieving detection limits as low as 100 copies of viral RNA per ml of viral transport medium [11].

**Figure 1:**
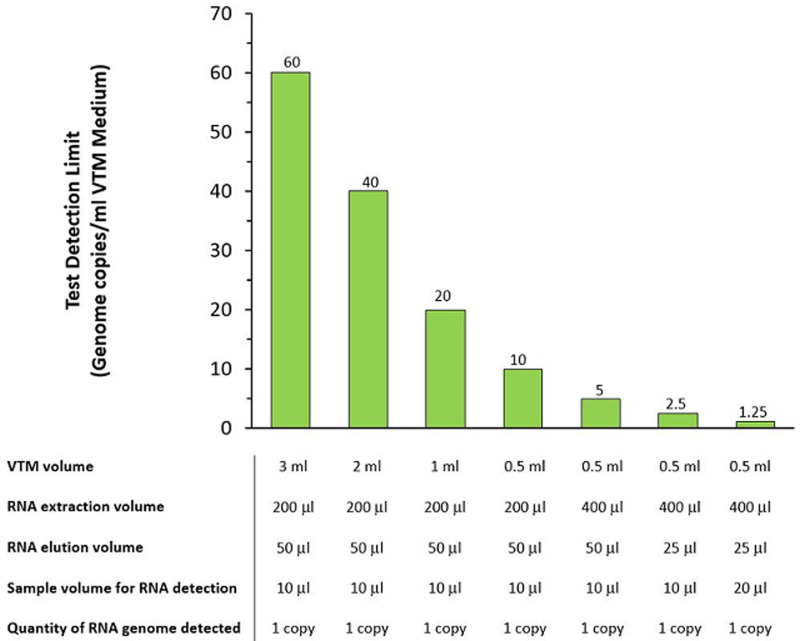
improving the detection limits of COVID-19 diagnostic workflows

**How the diagnostic sensitivity of COVID-19 workflows can be improved:** by taking into account all the steps of a workflow, the test sensitivity can be vastly improved by considering volumetric factors, which do not require any changes to the hardware, software, or chemistry underpinning the diagnostic protocols (Figure 1). The most obvious volumetric adjustment within a COVID-19 workflow would be to simply reduce the volume of the viral transport medium. Lower volumes (< 1ml) would not only ease the logistics of sample handling, transportation and storage of clinical samples, but also increase the working concentration of the viral RNA. There is no clear precedence within the literature, nor a strong scientific case, for the use of 1 to 3ml of viral transport medium during COVID-19 testing. A higher working RNA concentration would mean that smaller sample volumes could be carried forward to stage 2 or even directly to stage 3 which, in turn, would reduce the amount of reagent required for both stages.

A 200 μl volume of the patient sample is typically taken at stage 1 and used for RNA isolation during stage 2. Sensitivity could be boosted further by increasing the volume of patient sample. Greater carry-over of RNA into the later stages of the workflow would increase the likelihood of nucleic acid detection. For elution of the viral RNA, the final volume is usually 50 μl. Decreasing the elution volume would increase the RNA concentration and, in turn, allow a greater quantity of RNA to be carried over into the final stage of the workflow.

For the final stage, the use of highly concentrated master mixes would help to maximise the amount of RNA material that is analysed. A typical RT-qPCR reaction is performed in a 10 μl or 20 μl volume using a 1.6-fold master mix. A more concentrated master mix, say at 5-fold, would increase the amount of RNA material by at least 1.5-fold. Consequently, a concentrated master mix would also permit smaller reaction volumes to be set-up in the case of RT-qPCR which would greatly speed up run-times. The simple, yet subtle volumetric changes proposed at each stage of the workflow may at first seem insignificant but taken together the overall improvement in detection limits (RNA copies per ml) over the entire workflow could be enhanced by at least one order of magnitude.

## Conclusion

The issue of test sensitivity is particularly relevant in the early and late stages of the COVID-19 disease when viral RNA loads are poor [25]. Even simple volumetric adjustments, as highlighted within this article, have the potential to vastly improve the detection limit of diagnostic tests. Thus, we would encourage researchers to adopt a holistic approach, in which all the steps of a COVID-19 diagnostic workflow, are carefully scrutinised, particularly those upstream factors at the viral sampling and pre-analytical stages.
